# Diphtheria; Its History, Causes, Symp-Prognosis, Treatment and Sequelæ

**Published:** 1882-03

**Authors:** N. S. Davis

**Affiliations:** Prof. Principles and Practice of Medicine; Chicago


### CHICAGO MEDICAL JOURNAL & EXAMINER.

VOL. XLIV.—MARCH, 1882.—NO. 3.

### Original Lectures.

ARTICLE I.

### DIPHTHERIA: ITS HISTORY, CAUSES, SYMPTOMS, PROGNOSIS, TREATMENT AND SEQUELÆ. A Lecture in the regular course on Practical Medicine, in the Chicago Medical College (the reporter's manuscript carefully revised by the author), by N. S. DAVIS, Prof. Principles and Practice of Medicine, February, 1882.

GENTLEMEN: The word diphtheria, as used to designate a particular form of disease, is of recent origin, having been first applied to that purpose by Bretonneau in a valuable paper laid before the French Academy of Medicine, in 1821. While the name is thus modern, the disease has been recognized and described, with varying degrees of accuracy, from a very early period in medical history. The Grecian writers alleged that the disease originated in Egypt, and called it "*Malum Egypticum*," in the days of Homer and Hippocrates. An epidemic of the disease in Rome was recognized and described by Macrobius in the year 380, A. D. From that time to the middle of the sixteenth century, I find but few allusions to the disease. At the latter date, 1557, it appeared in Holland as an epidemic; in Germany, in 1650; in France and Italy, in 1749; and in England, from 1760 to 1769. While the descriptions of all these earlier epidemics are sufficiently accurate to render it certain that the writers were, for the most part, describing the disease now called diphtheria, it is equally evident that they often confounded it with scarlet fever and various forms of sore throat. Perhaps the most accurate of the early records is, "An Account of the Putrid Sore Throat," as it prevailed in London, by Dr. John Fothergill, published in 1769. The earliest account we have of this disease in America was written by Dr. Douglass, of the Massachusetts Colony, in 1736. It undoubtedly prevailed in New York in 1771, and was pretty clearly described by Dr. Samuel Bard. From that time to 1831 we find nothing in the medical literature of our country which could be regarded as applying to true diphtheria, although it was more or less prevalent on the continent of Europe, and was being carefully investigated by Bretonneau, at Tours, from 1818 to 1821.

Dr. John Bell, of Philadelphia, alludes to the prevalence of an epidemic sore throat in that city in 1831, which was evidently true diphtheria. In 1856 the disease prevailed with great severity in San Francisco and the adjacent counties in California, and during the next two or three years epidemics appeared in various parts of New England, New York, and a large number of the Middle and Western States. According to Dr. L. N. Beardsley, of Milford, Connecticut, the disease commenced in the adjoining town of Orange, among the scholars attending a select school, and with such severity that "fourteen cases out of fifteen, of those who were first attacked, proved fatal." In April, 1858, it made its appearance in Albany, N. Y., and caused 167 deaths during the next eight months. The disease began to attract attention in this city in 1858, and prevailed with considerable severity for three or four successive years.

During the same period of time, it showed itself more or less prevalent in almost every inhabited district of country from the Atlantic to the Pacific Ocean, and from the Lakes to the Gulf of Mexico. It prevailed in localities the most diverse in all their local conditions. Elevated, dry, thinly populated rural districts were visited as freely, and often as fatally, as the lowest alluvial valleys, or the most densely populated cities. It presented every gradation of severity, from fifteen deaths in sixteen attacks, as reported by Dr. Beardsley, of Connecticut, to only four deaths in one hundred and thirty-three attacks, as reported by Dr. Wm. L. Wells, of Milwaukee, Wis. For additional facts regarding the prevalence of this disease during the years intervening between 1858 and 1860, and earlier, I refer you to an interesting report on the topography and epidemics of New York, by Dr. Joseph M. Smith, in the Transactions of the American Medical Association, Vol. xiii, p. 251, 1860. From that period to the present it is not probable that the disease has been entirely absent from all parts of the country for a single year. We find it occupying a place of more or less prominence in nearly all the annual tables of mortality in our cities, and accounts of its prevalence in some of the country districts have come to us every year. It seldom prevails in the same rural district more than two or three years in succession, without a period of exemption. And in the larger cities it presents its distinct waves of increase and decrease. For instance, in Philadelphia the number of deaths from diphtheria each year for eight successive years was, in 1872, 141; 1873, 106; 1874, 181; 1875, 656; 1876, 708; 1877, 458; 1878, 464; 1879, 321. In this city (Chicago) and in New York, the statistics of mortality indicate the same wave of increase in 1875, culminating in 1876, and receding through 1877–78–79.

*Causes.*—The predisposing causes or circumstances that appear to favor diphtheria are, childhood and youth; dampness, with frequent changes in the thermometric conditions of the atmosphere; overcrowding of houses and consequent lack of ventilation; the presence of the products of the decomposition of organic matter, whether animal or vegetable; and the want of attention to personal cleanliness and domestic hygiene.

While it is true that, during the epidemic prevalence of diphtheria, persons have been attacked at all periods of life, from infancy to ripe old age, very much the larger number of cases occur in childhood. Of the 133 cases reported by Dr. Wells,

107 were in children and 26 in adults. Of the latter, one was 63 years of age; while the great majority of the former were between the ages of two and ten years. Dr. Willard, of Albany, in giving an account of an epidemic in that city, reports 179 deaths, of which only *three* were adults, all the remainder being children, most of whom were under twelve years of age. From the statistics of the severe epidemic prevalence of this disease in England from 1857 to 1860, it would appear that more than 85 per cent. of all the deaths were of children under the age of fifteen years. It is probable that seventy-five per cent. of all the cases of diphtheria occur in childhood under twelve years of age. And as one attack of the disease does not destroy the susceptibility of the system to subsequent ones, there must be something in the conditions of childhood that acts the part of a predisposing influence.

While isolated cases of diphtheria occur in particular houses or circumscribed localities at all seasons of the year, and epidemics have occurred in all varieties of climate, yet it remains true that the disease, especially in epidemic forms, prevails much the most frequently and severely within the temperate zone, and during the spring and autumn months, when atmospheric conditions are most variable. That overcrowding of the population, as in the tenement houses in our cities, with neglect of ventilation, and the accumulation of vegetable and animal matter from want of sewerage and cleanliness, act as strongly predisposing influences, is abundantly shown by the behavior of the disease in all our large cities during its special periods of prevalence from 1856 to 1860, from 1864 to 1867, and from 1875 to the present time. For instance, in New York City, during the three months ending September 30, 1881, the whole number of deaths from diphtheria was 545; of which 405 took place in tenement houses, leaving 140 to occur in all other dwellings. During the last five months of the year 1877, there were reported to the health officer of this city (Chicago), 162 deaths from diphtheria, occurring in 122 dwellings. The health officer caused each of these houses to be very thoroughly examined by an expert plumber and sewer builder, whose report showed 13 of these houses to be in excellent sanitary condition in every respect; 14

faulty from insufficient ventilation only; 19 from insufficient ventilation and uncleanliness; 24 from insufficient ventilation and uncleanliness, both of persons and premises; and 52 from defective sewerage and plumbing.

Facts of similar import may be gathered in connection with the prevalence of the disease in all large cities; and they justify the conclusion that in such aggregations of population, those persons and families who live in poorly ventilated, uncleanly, and imperfectly sewered houses and premises, yield a larger proportion of victims of diphtheria, than those in good sanitary surroundings. But, as I have already pointed out to you in previous lectures, this conclusion is equally, or even more, applicable to the prevalence of typhoid, typhus, and all other acute general febrile affections of kindred type. And, consequently, the only legitimate deduction from the facts is, that the diminished power of vital resistance from impaired tone of health caused by living in bad sanitary conditions, causes a more ready yielding to the influence of the essential cause or causes of diphtheria.

On the other hand, in villages and country districts, the disease has prevailed in its epidemic form with as much severity and fatality, in proportion to the population, as in the most densely populated cities, and in such districts it has shown little or no preference for the poor or uncleanly, but has invaded dwellings kept in the most perfect sanitary condition, and rural districts usually deemed most healthy. For instance, Dr. William C. Wey, of Elmira, N. Y., after carefully noting the rise, progress and decline of an unusual prevalence of diphtheria in that place during the years 1877–78–79–80, says: "In the epidemic which has so severely visited Elmira, the questions of filth and water-supply from unclean sources, as means of inducing and spreading the disease, have been carefully considered. In some cases the water supply has been found corrupted; in many the general surroundings have been unsanitary, and the facilities and comforts of the sick and attendants limited and unsatisfactory. As a matter of course, great mortality has followed in the train of neglect and poverty. In other cases the water supply has come from pure sources, the sanitary conditions of the people and their manner of living has been faultless, the utmost watchfulness has been exercised to maintain rigid non-intercourse with seats of the disease, and yet, in spite of care, and as if in defiance of it, the affection has appeared with as much malignity as in places of human crowding and disregard of sanitary precautions."

These are the statements of one of the most experienced and intelligent practitioners in that State. To precisely the same import are the facts given by Dr. N. B. Bailey, of Brewster, Putnam County, N. Y., in an account of an epidemic of diphtheria that prevailed in that place during the years 1877–78, published in the same volume of Transactions from which I have just quoted. My own personal observations lead to the same conclusions. I have met with the disease here, in this city, in all grades of severity, among both rich and poor, in the stateliest mansions of luxury, and in the most narrow, dark, damp and uncleanly hovels of poverty and vice. It has visited, from time to time, almost every county in this and neighboring States, and has proved as malignant and fatal, in proportion to the population, on the open plains, and thinly populated, healthy, rural districts in Northern Illinois, Wisconsin and Minnesota, as in the most unsanitary wards in the city of Chicago. At most, therefore, bad sanitary conditions can only be regarded as predisposing influences, and we must look in other directions for the efficient cause or causes on which the disease depends.

Many of the most eminent observers and writers of the present time represent the essential cause of the disease to be a specific contagium vivum, or organic germ, which has been shown to exist abundantly in the diphtheritic exudation or membrane, and in the epithelial layer of the membrane lining the fauces and other parts affected by the local manifestations of disease. By some, this organic germ is claimed to be the "Oidium Albicans," a fungus, consisting of sporules or micrococci and mycelium. In 1868, Buhl, Hueter, and Oertel discovered in the diphtheritic membranous formations, and in the mucous membrane covered by them, various species of bacteria, the most important of which were an exceedingly minute spherical variety, called by Cohn micrococcus, and the bacteria termo, or rod-shaped bacteria. By Oertel, and many others, these minute organisms are regarded as the essential cause of the diphtheritic disease, and they claim to have produced well characterized diphtheria in animals by inoculation with portions of the membrane from the fauces.

On the other hand, the results of the experiments of Burdon-Sanderson with filtered liquids; the failure of Trousseau and Peter to induce the disease in themselves by the very free application of the membranous substance to their own fauces; and the entire failure of Curtis and Satterthwaite, and W. C. Wood and Formad, to induce the disease in animals by repeated and carefully executed inoculations with diphtheritic matter, go far to disprove the conclusions of Oertel and his followers. And if we add to these the further fact that every variety of germs found either in the membranous exudations of diphtheria, or in the blood and tissues of diphtheritic patients, have also been found in the miguet or curdy exudations upon the mucous membrane of the mouth and fauces in young children, in the white exudations upon the tonsils in the last stages of consumption and other wasting diseases, and in the exudations that sometimes appear on different parts of the mucous membrane in typhoid, typhus, and other low forms of fever, we shall find it much more in accordance with sound principles of reasoning to conclude that these minute organic forms, called bacteria, micrococci, etc., are simply accompaniments, if not products, of certain degenerative organic processes that take place, to a greater or less extent, in all the acute febrile and inflammatory affections of an asthenic type. If we adhere impartially to well ascertained facts, we must admit that diphtheria often makes its appearance in families, asylums and schools, as well as at the beginning of epidemics, under such circumstances that it is impossible to trace it to any form of communication with previous cases, either in the same localities or elsewhere. In other words, it is capable of spontaneous development, and consequently does not depend for its production and spread upon any specific contagious germs or virus generated in the bodies of the sick. I have seen many cases illustrative of this fact; and the same is strikingly exemplified by the outbreak of the disease in Brewster, N. Y., as described by Dr. Bayley, in 1878.

The same adherence to simple facts, however, compels us to admit that in very many cases the disease appears to spread by an infection capable of contaminating clothes and furniture, and of being carried by them from family to family, and from one locality to another. Oertel recognizes both these series of facts, and claims that when it develops spontaneously, it is from some organic *miasm;* and hence he includes diphtheria in his class of "miasmatic contagious diseases." The fact that this disease usually prevails as an epidemic, commencing often without any traceable communication with previous cases or known contagious influence, and attacking, simultaneously, members of families in different parts of a city, village or rural district, who have neither had any communication with each other nor with any known common source of infection, would indicate that its essential cause consists in some special condition of the atmosphere, which the older writers called an epidemic constitution. Its nature will remain unknown until more systematic and continuous observations are made and recorded, concerning all appreciable conditions of the atmosphere in direct connection with records of the prevalence of acute general diseases. When this is done through a series of years, with the accuracy now attainable by the aid of physics, chemistry and microscopy, we shall have such elements for comparison as will throw light upon this and many other obscure questions connected with the etiology of diseases.

*Symptoms.*—For the clinical study of diphtheria I shall group the various cases met with under three heads—namely: the simple, the croupous, and malignant. In the first group I shall include all the cases that present so moderate a degree of severity as to pass through their successive stages with a natural tendency to convalescence. In the second group I shall include all those cases in which the local inflammation invades the larynx and trachea. In the third group will be included all such cases as, by the gravity of the general morbid conditions, or the severity of the naso-pharyngeal and glandular inflammations, tend strongly toward a fatal result. The majority of cases of simple diphtheria are developed gradually; the patient feeling for one, two or three days a gradually increasing sense of weariness; indisposition to mental or physical activity; vague or ill-defined pains in the head, back and limbs; with indifference to food. Then the face becomes a little flushed, the lips dry, the expression of countenance dull, the pulse moderately accelerated, with an increase of one or two degrees of temperature, and a more decided sense of weakness; and, in addition to these symptoms of a moderate general fever, there is observable a little undue fullness behind and beneath the angle of the jaw, with some feeling of stiffness and soreness in swallowing. On examining the fauces at this stage, you will find the mucous membrane covering the tonsils, arch of the palate, and portions of the pharynx, presenting a tumefied and dark red appearance, with some spots of white, or yellowish white, membranous exudation closely adhering to it, together with some degree of swelling of the tonsils and neighboring lymphatic glands.

In a smaller number of the cases belonging to this group, the attack is more abrupt, and accompanied by chilliness, or even a decided chill, followed by a more active general fever, but the same local symptoms just described. The symptoms thus begun usually gradually increase during the succeeding three or four days. The patches of membrane on the inflamed surface of the fauces increase in number and size, until, in many cases, they coalesce and cover nearly the whole surface, and extend with the inflammation into the pos erior nares. During the same time the tonsils and lymphatic glands also increase in size, impeding the free opening of the mouth, and rendering deglutition more difficult. The fauces also become troubled with an excess of tenacious mucus, which in young children often causes much rattling in the throat and some cough. The urinary secretion is moderately diminished, and in a small proportion of cases contains some albumen, and the bowels usually remain quiet, unless disturbed by laxative medicine.

This class of cases usually reach the climax of activity, in both general and local symptoms, in from three to five days after the first development of local symptoms. The swelling of the glands of the neck and parts within the fauces ceases to increase; the membranous exudation soon appears more yellow and shows signs of loosening or disintegration; the saliva or mucus in the mouth and fauces becomes more opaque, more easily dislodged, the breath more offensive, and generally some discharge from the nostrils. While these local changes are taking place, the general febrile symptoms also diminish; and in the mildest variety of cases, by the end of the first week, the temperature has returned to the natural standard, the pulse becomes soft and weak, but natural in frequency; the cutaneous and urinary secretions natural, and the membranous exudation and swelling, both in the fauces and lymphatic glands, disappear, leaving the patient fairly convalescent, yet much debilitated. In the more severe cases belonging to the first group, the morbid phenomena reach their climax in the same length of time, and the same subsequent changes take place, but the subsidence of the glandular swellings and the disintegration of the membranous exudations progress slower, and are accompanied by more copious and troublesome discharges from the mouth and nostrils, and greater offensiveness of the breath. The patient also exhibits more dullness, with paroxysms of restlessness, especially when the fauces and nostrils become obstructed by the mucous or muco-purulent discharge, as is apt to be the case in infants and young children. The pulse becomes more weak, the bodily temperature returns more slowly to the natural standard, although the skin and extremities may even feel unduly cold, and the patient is longer troubled with difficulty of deglutition, and more tendency of food and drink to regurgitate through the nostrils. Yet, in nearly all of these cases, the disease completes its course, and convalescence is established by the middle or latter part of the second week. In some, however, the breaking up and disappearance of the false membrane is accompanied and followed by superficial ulcerations in the tonsils and other parts of the throat; and, in a smaller number, one or more of the inflamed lymphatic glands suppurate, forming abscesses in the neck. These occurrences may postpone the establishment of convalescence until some time during the third week from the commencement of the attack.

The second group of cases, including all those in which the diphtheritic inflammation invades the larynx, will be presented to you under two aspects: one, in which the inflammation enters the larynx apparently by extension from the pharynx, and generally manifests itself first between the fourth and seventh days after the commencement of the disease, or even after convalescence has fairly commenced; the other, in which the inflammation attacks the larynx primarily, giving rise to hoarseness of voice, stridulous breathing and croupal cough, from the beginning of the patient's sickness. In both, the general symptoms are the same as in ordinary diphtheria.

In all the cases in which the local disease develops in the larynx by extension from above downward, and does not commence until several days after the beginning of the general diphtheritic disease, there can be no difficulty in making the diagnosis. But when the larynx is invaded coincidently with the beginning of the sickness, there is often much difficulty in keeping a clear line of distinction between the diphtheritic disease and the ordinary sporadic pseudo-membranous laryngitis. And many writers of the present day regard them as identical, and do not attempt to distinguish the one from the other. In all the cases that have come under my observation, however, the diphtheritic laryngitis has been accompanied by some redness and swelling of the tonsils and other glands in the neck ; a soft, weak pulse ; more dullness of expression, and earlier symptoms of exhaustion. In all these cases the inflammation in the larynx is accompanied by a rapidly increasing exudation, which solidifies into a thick, firm layer of false membrane over all the interior of the larynx, the cartilages at the opening of the glottis, and often downward through the trachea and into the larger bronchial tubes. The voice becomes early suppressed, the cough rough, stridulous and suffocative ; the breathing difficult, and accompanied by a tight, wheezing sound in the neck at first, but subsequently accompanied by mucous rattle. To these local symptoms of direct obstruction in the larynx, there is added a soft, quick, weak pulse, somewhat purplish or leaden color of the lips, and fullness or bloating of the face ; coolness of the extremities, with moderate increase of temperature in the head and trunk of the body ; drowsiness, with temporary paroxysms of restlessness and tossing ; often difficulty of deglutition, and scantiness of urine. In very severe cases, the dyspnœa and rattling in the throat and larynx increases every hour, with frequent paroxysms of choking, strangling cough, during which more or less of a thick, ropy mucus is forced out, containing shreds of the false membrane ; after which, for a brief time, the breathing is easier. But the obstruction soon accumulates again, causing the sense of suffocation and struggling for breath to be renewed, until the imperfect oxygenation and decarbonization of the blood render it no longer capable of sustaining the sensibility of the brain and nervous centers, when the patient becomes somnolent or stupid, the breathing frequent, very difficult, and accompanied by coarse mucous rattling in the air passages; blueness of the lips; coldness and blueness of the extremities; a small and very weak pulse; and finally, relaxation of the sphincters, a general clammy sweat, and death from asphyxia. The fatal result is reached in some of these cases in five or six hours; in a much larger number, however, it is deferred from two to five days. If the tumefaction of the parts within the larynx and the membranous formations are not sufficient to destroy life in from three to five days, the latter begin to loosen and disintegrate, and in the paroxysms of coughing more shreds and patches of the membrane are dislodged and thrown out with a more opaque, muco-purulent expectoration.

The tightness and constriction in the breathing diminishes; the color of the skin and expression of countenance improve; the pulse becomes slower; the mind more active; and in three days, or from seven to nine from the commencement of the laryngeal trouble, all bad symptoms have disappeared, leaving the patient convalescent, but much debilitated. In some cases of diphtheritic laryngitis, the membrane is detached and thrown out, in the severe paroxysms of coughing, in large pieces, presenting, when inflated, more or less of a complete model of the interior of the larynx. Many years since I saw a case in consultation with my colleague, Dr. Hollister, in which the patient, a boy aged seven years, in a violent paroxysm of coughing, expelled a perfect tubular cast of the larynx and trachea, measuring seven inches in length to the bifurcation, and extending beyond to the primary division of the bronchial tubes, having thirteen divisions on one side and eleven on the other. The expulsion was followed by a great degree of immediate relief; but, as frequently happens, the relief was only temporary. Fresh exudations took place on the inflamed membrane, and, extending lower into the bronchial tubes, renewed the dyspnœa, and proved fatal before the end of the next twenty-four hours.

In the third, or malignant group of cases, the onset of the attack is generally abrupt, and attended by appearances of a chill or cold stage, which is followed by a more rapid rise of temperature of the body; a more rapid development of inflammation and swelling, both in the fauces and glands of the neck, often causing in a very few hours great difficulty of deglutition, inability to open the mouth widely, and the speedy formation of a thick, tough, yellowish membrane over the whole arch of the palate, tonsils and pharynx. The pulse is frequent, soft and weak; breathing noisy from the existence of tenacious mucus in the throat and nostrils, and more frequent than natural; the expression of countenance dull, with a dark or purplish flush; extremities often cool, with leaden color under the nails; urine scanty, and often containing some albumen; and the mind inclined to drowsiness, except in momentary paroxysms of restless tossing, or of efforts to clear the mucus from the throat. Sometimes the attacks of this variety are so severe, and the tumefaction of the tissues within and behind the angle of the jaw so great, that the blood is obstructed in its return from the brain, causing stupor, inability to swallow, extreme frequency and feebleness of pulse, and death in from twelve to eighteen hours after the beginning of the attack. In other cases, the obstruction to the cerebral circulation is less, and life is prolonged from one to five days. In such, during the second day, the inflammation and exudation extend into the nostrils posteriorly, and sometimes into the Eustachian tubes, and even into the middle ear. During the third and fourth days the false membranes begin to disintegrate, the mucus in the fauces and nostrils becomes more abundant, more opaque or muco-purulent, decidedly offensive, and gives occasion to much noise and difficulty of breathing. The inflamed mucous membrane also shows commencing ulceration, and in many cases gangrene. The patient loses strength rapidly, and usually dies from complete exhaustion before the end of the fifth day.

I have now given you a summary of the more important symptoms of the different grades of diphtheria. You observe that it is a disease varying greatly in its degree of severity in different seasons, and in different cases the same season. So true is this, that I have in some years attended a large number of cases with less than two per cent. of deaths; while in other years, with no greater numbers, the proportion of croupal and malignant cases was so great that the deaths averaged from ten to fifteen per cent. It is not rare that, in the more severe epidemics of this disease, all the children in a family are destroyed within a few days. I remember being called to visit, in consultation, a Scandinavian family in the northwestern part of the city, where I found three children lying side by side on the same table, dressed for burial, and a fourth one dying, leaving only the nursing infant in its mother's arms.

Thus far I have spoken of the diphtheritic membranous exudations as appearing only in the throat and parts in immediate connection with it. The same, however, may attack the vagina and vulva, the lips, the wing of the nose, the conjunctiva of the eye, and any sore or raw surface in any part of the body. I saw one well-marked case, in which a thick layer of false membrane covered one-third of the upper lip, accompanied by considerable tumefaction; and another in which a lady, recovering from an extirpation of a cancerous tumor from one breast, while there was still a healthy granulating surface unhealed, was attacked with the ordinary general symptoms of diphtheria, accompanied with a moderate degree of inflammation and exudation in the fauces. Simultaneously with the appearance of the latter, the uncicatrized surface on the breast became completely covered with a thick layer of false membrane, which remained about three days, and as it disintegrated and disappeared, it was accompanied by an abundant sero-purulent and offensive discharge, with a complete destruction of all the previously healthy granulations. After the diphtheritic disease had disappeared, the raw surface on the breast gradually resumed a healthy appearance, and subsequently progressed to complete cicatrization.

*Diagnosis.*—From catarrhal sore throat, diphtheria is distinguished by the character of the general fever; the coincident inflammation of the mucous membrane of the fauces and tonsils, with tumefaction of some of the lymphatic glands near the angle of the jaws; and still more by the appearance of more or less diphtheritic exudation on some part of the inflamed structures. The diagnostic differences between diphtheria and ordinary sporadic croup or active pseudo-membranous laryngitis, I pointed out when giving the symptoms of the croupous variety of diphtheria, and need not repeat them. From scarlet fever, diphtheria is distinguished by the much less sudden and severe onset of the fever, the presence of diphtheritic membranous exudations, and the absence, generally, of any exanthematous eruption upon the skin. In some epidemics of diphtheria quite a proportion of the cases will be accompanied by a moderate amount of a fine red exanthematous rash, causing the surface to much resemble mild cases of scarlet fever. But the milder grade of general fever, and the coincident existence of white patches of diphtheritic membrane in the fauces, will usually enable the practitioner to keep the diagnosis correct. It has undoubtedly happened, however, when both these general febrile diseases were prevailing in the same community, that they have manifested a disposition to commingle the characteristic symptoms of both in the same patient, thereby causing doubt and sometimes controversy concerning the diagnosis. It is more proper to regard such cases as presenting the combined or simultaneous presence of the causes of both diseases, in the same manner as we recognize the coincident action of the causes of typhoid and periodical fevers, producing what has been styled typho-malarial fever.

*Prognosis.*—The prognosis has been pretty fully indicated by the clinical history I have just detailed to you. All the milder cases tend toward spontaneous recovery in from seven to fourteen days. The more malignant and the croupous groups of cases manifest a strong tendency to end in the death of the patient, and are always productive of a high ratio of mortality.

*Pathology.*—I regard diphtheria as a general febrile affection, arising from some cause or combination of causes by which the properties of the blood and of the organized structures are so changed as to render the fibrin more disposed to solidify or coagulate than natural, and to lessen the tone and contractibility of the muscular structures, with special tendency to develop asthenic inflammation of greater or less severity in the mucous membrane of the throat and adjacent lymphatic glands. That the general disease is one of a typhoid or adynamic character, and the local inflammations asthenic, is proved by the generally soft, compressible pulse, universal muscular weakness, liability to syncope from moderate exertion, and the aplastic character of all exudations. By the latter I mean the uniform tendency of all the membranous exudations, however thick or tough they may be, to undergo degeneration and dissolution, never taking on permanent organization or becoming a bond of union by adhesively uniting surfaces that may be in contact with each other. This view is further corroborated by the frequent occurrence of muscular paralysis as a sequel of the disease.

*Treatment.*—From these views of the pathology of diphtheria I deduce four well defined, rational indications to be fulfilled by treatment: First, to arrest the further infection and deterioration of the blood. Second, to improve the general tonicity of the tissues by increasing the vital affinity. Third, to sustain the nutritive and excretory functions as near their natural condition as possible. Fourth, to mitigate the violence of such local inflammations as may exist in each individual case. To fulfill the first of these indications, the chief reliance has been placed on the internal use of chlorine, bromide, iodine and their salts, such as the chlorates of potassium and sodium ; and to this have been added more recently, the sulphurous acid and the sulphites of sodium and calcium, the benzoate of sodium, the sulpho-carbolate of sodium, and the permanganate of potassium. Of these, I think the aqueous solution of iodine, the chlorate of potassium and the benzoate of sodium are the most important. To fulfill the second indications, I rely principally upon a judicious use of quinia, iron, strychnia, pure air and nourishment; even when temporary stimulants are needed, carbonate of ammonium and camphor are the most reliable. Many recommended strongly the use of some one of the alcoholic class of drinks, and mention the extraordinarily large doses borne by diphtheritic patients without intoxicating effects. So far is this from affording a reason for their use, that I should construe it in the opposite direction. Both the general susceptibility of the tissues and the sensibility of the nervous system are blunted or below the normal standard, and consequently, anæsthetics like alcohol, are neither indicated nor readily responded to when given. The same principle or therapeutic rule applies here, that I explained more fully when speaking to you in relation to the treatment of typhoid fever.

If you can succeed well in fulfilling the first and second indications as now explained, the fulfillment of the third follows as a necessary result. Yet, when called early, and you find the skin hot and dry; urine scanty; tongue coated; and bowels inactive, you can give a small alterative dose of calomel with bicarbonate of sodium, every three or four hours, until three doses are taken; and if the bowels do not move in three hours after the third dose, give a mild laxative, and it will generally produce a favorable effect. The action of the skin and kidneys may be further sustained by suitable doses of spirits of nitrous ether and liquor ammoniæ acetatis. To fulfill the fourth indication, namely—to lessen the severity of the local inflammation in the fauces, air passages, and glands of the neck, a great variety of local applications have been used. During the severe epidemic in this country, occurring between 1856 and 1864, nitrate of silver in all gradations of strength, from the solid stick to a solution of 0.33 grams (grs. v.) to 30.0 cubic centimeters (fl. ℥j.) of water, was extensively and perseveringly used locally, with the expectation of arresting the membranous exudation and of limiting the extent of the inflammation. After an abundant experience, its use was abandoned by nearly all the more accurate observers as either useless or positively injurious. Applications of strong solutions of sulphate of copper, tincture of iodine, and tincture of the chloride of iron, were tried with no better results, and the profession generally had come to regard local applications of any kind as a matter of secondary importance, until Oertel and others again promulgated the doctrine that diphtheria is primarily a local disease, produced by the direct action of bacterial germs on the mucous membrane of the fauces and air passages, and through which they entered the blood, and secondarily, produced general infectious fever. Under this teaching the early and thorough local application, not of caustics, but of strong antiseptics or germicides, as carbolic, salicylic, benzoic and sulphurous acids, were brought prominently to the notice of the profession, and received the unqualified commendation of many practitioners. During the past ten or twelve years diphtheria has prevailed with average severity in most of our large cities and in many country districts, affording abundant opportunities for testing the virtues of this class of remedies. And, aided by the coincident extravagant ideas in regard to the uses of antiseptics in the practice of medicine and surgery generally, they were enthusiastically applied in every form, and every degree of strength; in solution, with the swab and syringe; in spray, with the atomizers; and in vapor, by inhalations. As might be expected, the results, as reported at the various medical society meetings and through the medical press, have been varied. As a general rule, those who met the disease in a mild form reported great success. Those who met the disease in its more severe and malignant aspects, reported the usual ratio of mortality, and pronounced the germicide treatment useless. A middle class of practitioners, like a member of the Illinois State Society, reported, with enthusiasm, that the thorough application of pretty strong solutions of carbolic acid had aborted every case that had come under his treatment *before* the inflammation and exudation had entered the posterior nares or the larynx. But unfortunately the disease had extended beyond these limits in so many cases before coming under treatment, that the actual ratio of mortality to the whole number of cases treated, was the same as usual.

So, gentlemen, if you will diligently examine the statistics of cases and mortality in all the cities and municipalities in which such statistics have been kept, for the past ten years, in which germicidal theories and practice have predominated, with the same class of statistics for the preceding ten years, you will find no evidence that such practice has resulted in diminishing, in any degree, the ratio of mortality below that of the former decade. Having already stated to you that I regard diphtheria as primarily a general febrile affection, developing certain local inflammations of peculiar character during its progress, and having now explained the several object, to be accomplished in its treatment, it only remains for me to indicate more definitely which of the remedies mentioned for the several purposes I deem best, and their mode of use as adapted to the several stages of the disease. In the milder cases of simple diphtheria, very little medication is either necessary or proper. For such, I direct a diet of milk and farinaceous articles, rest, as fresh good air as possible, a moderate, comfortable temperature of the room, and the following prescription for medicine :

| | | | |
|---|---|---|---|
| ℞ | Potassii Chloridi.................10.0 grams. | | ʒjjss |
| | Acidi Muriatici................ 4.0 c. c. | | ʒj |
| | Tincturæ Belladonnæ....... 10.0 c. c. | | ʒjjss |
| | Aquæ...........................260.0 c. c. | | ℥viii |

Mix. Give from two cubic centimeters (fl. ʒss.) to eight (fl. ʒjj), or from half a teaspoonful to a dessert spoonful, according to the age of the patient, every two or three hours, without further dilution. The application of this solution to the fauces and throat, is made much more complete and easy by swallowing it, than by any process of swabbing, sponging or gargling; while its introduction into the system constitutes one of the best means for fulfilling the first indication for general treatment. The solution of the chlorate of potassium with the mineral acid, combines the properties of an efficient antiseptic and tonic, while the influence of the belladonna on the vessels of the mucous membrane and glands of the throat and neck tends to lessen both tumefaction and membranous exudation.

During the last thirty years I have treated very many cases of mild diphtheria, without any other medication than the use of the formula just given. If, at any time during its use, the effects of the belladonna accumulate sufficient to perceptibly dilate the pupils, the dose should either be diminished or given at longer intervals. In cases of greater severity, yet not positively malignant, I give the same formula in the same manner during the first three days after the commencement of the disease. If the patient has been previously healthy and well nourished, and the pulse and temperature rise pretty actively, with scantiness of secretions, I give in addition during the first day, an alterative dose of calomel at intervals of once in two or three hours until three doses have been taken ; and, if necessary, follow them by a mild laxative or warm water enema. After the bowels have moved, I direct a solution of iodine 0.33 grams (grs. v.) and iodide of potassium 2.0 grams (grs. xxx.) in 45 cubic centimeters (℥jss) of water, to be given in doses suited to the age of the patient, every six hours. During the same early stage, if the tonsils and glands behind and below the angle of the jaw commence to swell actively, I keep the external parts closely covered with cloths wet in an infusion of aconite leaves and chloride of ammonium, 30 grams (℥j) of the former, and 15 grams (℥ss ) of the latter to one litre (Ojj) of boiling water. When, from any cause it may be difficult to keep the wet cloths applied properly, the following liniment may be used instead:

℞ Olei Olivæ......................90.0 c. c. ℥jjj.
Olei Terebinthinæ............15.0 c. c. ℥ss.
Chloroformi.......................15.0 c. c. ℥ss.

Mix, and apply to all the external swollen parts every three hours, or often enough to keep the surface moist. If, under the remedial agents I have now mentioned the case progresses favorably, and when the time for the membranous exudations to begin to loosen and disintegrate comes, which is generally from the third to the fifth day, the breath and saliva do not become offensive, and the swelling of the glands does not increase further, there need be no essential change in the treatment except to lessen the frequency of doses as the disease declines, and an early convalescence will be reached. But if, at the stage just mentioned, the breath becomes offensive, the saliva more abundant, and mixed with more or less muco-purulent or sanious discharge from the throat and nostrils, with more dullness of expression and a softer pulse, I immediately exchange the chlorate of potassium and belladonna solution for the tincture of chloride of iron and quinine, given in moderate, but frequently repeated doses, and require more diligence in giving nourishment. The solution of iodine may generally be given with benefit two or three days longer. Under the influence of the quinine, iron, and simple nourishment, the patient will pass the crisis of the disease with only a moderate amount of ulceration and suppurative action in the inflamed membranes, and the general febrile symptoms will gradually decline until convalescence is established. In some of the more severe and malignant cases, the crisis of the disease is marked by great weakness, a more copious flow of offensive muco-purulent matter from the mouth and nostrils, and more extensive destruction of the inflamed structures by ulceration, and sometimes by gangrene. In such cases, I continue the use of the quinine and iron, and, in addition, give carbonate of ammonium and camphor, in moderate but frequently repeated doses, and add to what nourishment is taken by the mouth, the use of nutritive enemas. Unfortunately, in many of these cases, deglutition is so impaired that neither medicines nor nourishment can be swallowed in sufficient quantity to effect the needed support. Even in such, much can be done to sustain them until the throat begins to improve, by a judicious use of milk, beef tea, and other items of nourishment in the form of enemas, and most of the medicines required can be added to the enemas. Further support may also be given by inunction of cod-liver oil, in which may be suspended a small amount of strychnine. To a litre (Ojj.) of cod-liver oil may be added 0.2 grams (grs. iii) of strychnine. This may be well shaken and applied sufficient to anoint nearly the whole surface of the body three times a day. In the cases that present a strongly malignant aspect from the beginning, I give the quinine and tincture of chloride of iron, alternated with the carbonate of ammonium and camphor at once; and during the first twenty-four hours apply freely over the trunk of the body the cod-liver oil, holding in solution a small proportion of iodine; and, after the first day, the strychnine may be added in the proportion already stated.

During the last few years, I have used a solution of the benzoate of sodium as a substitute for the chlorate of potassium, and belladonna solution, in the early stage of the disease. Ten grams (ʒjjss.) may be dissolved in 120 cubic centimeters (fl. ℥iv.) of water; of which four cubic centimeters, or one teaspoonful may be given to an adult every two hours. It appears to exercise much influence in limiting the amount of the membranous exudation, and is particularly well adapted to the early stage of the more active sthenic class of cases. Again, in the second stage of the disease, if the muco-purulent discharge from the nostrils becomes copious and offensive, or irritating to the parts with which it comes in contact, it will do good to have nostrils syringed out freely at least twice in the twenty-four hours, with a weak solution of carbolic acid and sulphate of zinc or of permanganate of potassium. If an anodyne is required at night to aid in procuring rest, I know of none better than a powder containing the compound powder of opium and ipecac, 0.33 grams (grs. v.) and pulverized gum camphor 0.13 grams (grs. ii.) for an adult, and proportionably less for children.

I have now given you an outline of that course of treatment of the several stages and grades of severity of this disease, which has been, in my hands, the most beneficial to my patients, leading to the highest ratio of recoveries and leaving the smallest ratio of important sequelæ. One variety of cases, however, has been omitted from this outline, namely, the croupous or laryngo-tracheal. When the diphtheritic inflammation invades the larynx, whether primarily, with the beginning of the attack, or secondarily, by extension from the pharynx, I give, as early as possible, an emetic dose of the sub-sulphate of mercury, and repeat it at intervals of from two to six hours, until the stage of increasing exudation has passed. Thirteen centigrams (grs. ii.) of the sub-sulphate given in the form of powder, will generally produce prompt and free vomiting in children from three to five years of age. For older patients the dose should be increased, and for younger ones diminished. In the interval between the emetics, I give during the first twelve hours, a small dose of calomel and bicarbonate of sodium every two hours. To children from three to five years old, six centigrams (grs. i.) of calomel and twelve centigrams (grs. ii.) of the sodium, may be given at a dose. During the same period of time, a solution of lactic acid, 0.33 cubic centimeters (min. v.) to 30. c.c. (fl. ℥i.) of water should be frequently thrown into the fauces in the form of spray from an atomizer, and the liniment I have previously mentioned, containing oil of olive, oil of turpentine and chloroform, applied freely to the front and lateral parts of the neck externally. After the first four or five doses of the calomel and sodium have been given, they are omitted, and I give, instead, a solution of lactate of iron, 4 grams (ʒj.) to 120 cubic centimeters of water (fl. ℥iv.) of water, of which 2 c.c. (fl. ʒss.) or half a teaspoonful, may be given every two hours. If the invasion of the larynx has been secondary, several days after the commencement of the general disease, I omit the calomel and sodium powders, and commence the use of the solution of the lactate of iron, alternated with quinine, at once. In all other respects their management is the same as I have just indicated.

Sometimes a mild anodyne and expectorant influence is needed to lessen the violent spasmodic quality of the cough and aid in promoting rest—especially in the later stages of the disease. For such purpose I know of nothing better than an equal mixture of the compound syrup of squills and camphorated tincture of opium, given in doses suited to the age of the patient. If a judicious use of the remedies I have just detailed should fail to relieve the patient, and suffocation be impending, the only alternative is a resort to tracheotomy, which almost always affords a surprising degree of temporary relief, but is very generally followed by an extension of the inflammation into the bronchial tubes and the ultimate death of the patient. During all the treatment of the croupous cases the temperature of the room should be kept a little above the usual standard of healthy comfort, and the air constantly impregnated with aqueous vapor. Some place much reliance on the inhalation of the vapor of hot water, in which quicklime is undergoing the process of slacking. I have seen it perseveringly tried many times, but with very little effect. I regard it of far less importance than the lactic acid spray, and even less beneficial than the old-fashioned remedy, consisting of the free inhalation of the vapor from a hot infusion of hops in vinegar.

*Convalescence.*—Due attention should be given to the management of the period of convalescence from all grades of diphtheria. To secure a proper action of the skin and kidneys, and promote the renewal of a healthy tone and sensibility in the muscular and nervous structures, the patient should be kept much at rest; well protected from sudden atmospheric changes by flannel underclothes; judiciously supplied with fresh, dry air, and plain, nutritious, and easily-digestible food. In the convalescence from severe cases, the taking of a small dose of strychnine or nux vomica, with a soluble salt of iron at each regular meal-time, will be of much benefit, both in hastening the return of strength and in lessening the risk of paralysis.

*Prophylaxis.*—The best means of preventing the spread of diphtheria is to isolate, as far as practicable, all cases as they occur, and maintain essentially the same sanitary regulations as I mentioned for the prevention of typhus and typhoid fevers.

*Sequelæ.*—Congestion of the cortical texture of the kidneys, and general dropsy occasionally occur as a sequel of diphtheria, though much less frequently than after scarlet fever. For the treatment of such cases I refer you directly to the lecture on the sequelæ of the fever just named. The most frequent and troublesome of the affections that are liable to arise during the convalescence from diphtheria is some form of paralysis. In most cases it is limited to the muscles of the fauces and pharynx, and is only sufficient to simply give the voice a decided nasal quality, and make deglutition a little difficult; but is sometimes so complete as to render swallowing altogether impossible. During the prevalence of the disease in this city in 1858–9, I saw a case of this kind with the late Dr. J. A. Collins. The patient was a boy about eight years of age; and it was necessary to feed him liquid nourishment through a stomach-tube for two weeks, before he regained the power to swallow anything.

While the paralysis more frequently attacks the muscles of the fauces and throat, it may manifest itself in any one or more of the voluntary muscles in any part of the body and extremities. Or it may attack one set of muscles after another, until, like rheumatism, it has passed in succession over a large proportion of the voluntary muscles of the system. A case of this kind was brought to the Mercy Hospital two years since. It was in the person of a young man who had passed through a moderately severe attack of diphtheria, and recovered so far that he had begun light out-door work.

He first lost the power of speech and deglutition. In a few days these functions began to improve, when he lost all voluntary motion of the muscles of one side of his face and eye. Just as he was regaining control over these, the paralysis involved the muscles of both upper and lower extremities, and was so near complete that he could neither feed himself nor stand on his feet. It was at this stage of his disease that he was brought into the hospital. The paralysis following diphtheria, whether partial or complete, appears to be a simple loss of nervous force or muscular contractility, and is not accompanied by any inflammatory or febrile symptoms, or even local pain and soreness. It very generally tends towards recovery, and probably never ends fatally, except in very rare instances, when it attacks the muscles of respiration or of the heart. No case has terminated fatally or failed to recover, within the circle of my own practice. The only remedies necessary are rest, good air, nutritious and easily-digestible food, and muscular and nerve tonics.

Of the latter, we have none better adapted to these cases or more promptly curative than strychnine, citrate of iron, and the hypophosphites. To an adult or patient over fifteen years of age, I give a pill or capsule containing strychnine, two milligrams (grs. 1-30), and citrate of iron, thirteen centigrams (grs. ii), before each meal-time; and four cubic centimeters (fl. ʒi) of either the syrup of the lacto-phosphate of calcium, or of the compound syrup of the hypophosphite of sodium, calcium, and iron, half an hour after each meal. Of course, proportionately less doses must be given to younger children.

Under such management the patients usually make a good recovery in from one to four weeks. The daily application of mild currents of electricity or galvanism has also proved beneficial.

---

HOT WATER IN THE TREATMENT OF HÆMORRHOIDS.—Landowski (*Chl. f. Chir.* from *Jour. de Thérap.*) suggests hot sitz-baths in bleeding piles, together with enemata of hot water. These not only check the bleeding, but diminish the size of the turgescent tumors to a marked degree. In ordinary hæmorrhoids three sitz-baths per diem may be employed. In bleeding piles the baths should be more frequent, and the enemata should be given as hot as the patient can bear (usually about 104°). *Louisville Medical News.*

